# Not Only Length Matters! Impact of the Ileal Width on the Capacity of the Orthotopic Neobladder: The AADAPT Formula Tested on the Animal Model

**DOI:** 10.1016/j.euros.2023.10.003

**Published:** 2023-11-08

**Authors:** Filippo Annino, Thierry Piechaud, Robert Ghattas, Richard Gaston, Anastasios D. Asimakopoulos

**Affiliations:** a Unit of Urology, Azienda Toscana Sud-Est, San Donato Hospital, Arezzo, Italy; b Unit of Urology, Clinique Saint-Augustin, Bordeaux, France; c Independent Mathematician; d Unit of Urology, Fondazione PTV Policlinico Tor Vergata, Rome, Italy

**Keywords:** Cystectomy, Urinary diversion, Ileum, Small intestine, Urinary bladder, Bladder cancer

## Abstract

**Background:**

The capacity of a given shape of an orthotopic ileal neobladder (ONB) varies significantly, although the same length of preterminal ileum is utilised.

**Objective:**

To investigate the variability of the human ileal width and to create a mathematical formula that calculates its impact on the neobladder capacity.

**Design, setting, and participants:**

During 50 consecutive cases of robotic pelvic surgery, a segment of preterminal ileum was identified and the width was measured. A mathematical formula was created to calculate, for a given ileal length and width, the neobladder capacity and, for a given ileal width and neobladder capacity, the length of the (pre)terminal ileum to harvest. The accuracy of our model was tested on 28 pouches created by swine ileum.

**Outcome measurements and statistical analysis:**

The interindividual variability of the ileal width and its impact on the ileal neobladder capacity was investigated.

**Results and limitations:**

The mean hemicircumference of the human distal ileum is 2.43 ± 0.39 cm (range 2–3.5 cm). According to our geometric model and as confirmed in the swine model, an increase of 1 cm in ileal width increases the neobladder capacity by 85%. The Pearson correlation coefficient reported a strong positive relationship between the formula-calculated and effective volumes of the pouch (*r* = 0.97). Moreover, for the same target capacity, 1 cm of difference in the ileal width implies harvesting 20 cm less ileum. A lack of testing on humans and application only to spheroidal neobladders are the main limits.

**Conclusions:**

The ileal width impacts the capacity of the ONB. For a given type of ONB, no standard length of ileum should be harvested; instead, the length should be tailored to the width of the ileum for a given patient. Clinical studies are required to confirm our model.

**Patient summary:**

We demonstrated the variability of the ileal width among humans, and we provided a mathematical formula tested on swine that evaluates the impact of the ileal width on the capacity of the orthotopic ileal neobladder.

## Take Home Message

For a given type of ileal neobladder, no standard length of ileum should be harvested; instead, the length should be tailored to the width of the ileum for a given patient. Clinical studies are required to confirm our model.

## 1 Introduction

Since the early 1900s, surgeons have sought the optimal method to replace the original bladder when it must be removed because of either benign or malignant disease. Among the various options, an orthotopic neobladder (ONB) is the type of urinary diversion (UD) that most closely resembles the original bladder, both in location and in function [1]. In fact, an ONB allows for voluntary voiding, avoids the need for urinary control devices, and requires self-catheterisation only in a minority of patients [2], [3]. Patients with an ONB may present better quality of life than patients with other UD [4].

Several techniques for ONB reconstruction have been described. Their main objective is to obtain a spherical reservoir with adequate capacity. For each technique, a defined length of preterminal ileum is used. However, when analysing the maximum cystometric capacity (MCC) data of the same ONB, great variability is observed. For example, the 12-mo mean MCC of the Studer ONB varied between 321 and 530 ml in the evaluated studies [5], [6]. The 18-mo MCC of the modified W-shaped ONB varied between 310 and 720 ml [7], while the 6- and 12-mo MCC of the modified S-pouch ranged between 290 and 420 ml and between 350 and 570 ml, respectively [8]. At a mean follow-up of 27 mo, the MCC of the Bordeaux ONB varied between 200 and 553 ml [9], [10]. These variations in MCC were observed although the same surgeons used the same length of harvested ileum and the same ONB shaping.

The impact of the length of the ileal segment on the ONB capacity has extensively been studied. It is known that for a defined ONB shape, the variation in the length of bowel does not equate to a linear change in surface area and eventual volume, but it rather multiplies the surface area and the volume.

However, no study has evaluated the impact of the width (hemicircumference) of the ileum on the ONB capacity. The ileum, in fact, is a cylinder characterised by interindividual variability in its hemicircumference. As assessed by a recent magnetic resonance enterography study, concerning the distal ileum, great variability in diameter is observed, ranging between 11 and 31 mm [11].

The hypothesis of the current manuscript is that the variability in the hemicircumference of the distal (preterminal) ileum impacts the capacity of the neobladder, and consequently it should be taken into consideration before harvesting the ileal segment for the ONB reconstruction.

The aim of our study was to assess the interindividual variability of the ileal circumference. To evaluate the impact of this parameter on neobladder capacity, and to provide a mathematical formula that automatically calculates, for a given ileal length and width, the neobladder capacity and, for a given ileal width and neobladder capacity, the length of the (pre)terminal ileum to harvest.

## 2 Patients and methods

During 50 consecutive cases of robotic pelvic surgery, a 40 cm (stretched) segment of preterminal ileum at 25 cm from the ileocaecal valve was identified. With the aid of atraumatic robotic graspers, this segment was tethered against its mesentery, and its hemicircumference (both anterior and posterior) was measured at 10 cm from its ends. A sterile ruler placed perpendicular to the axis of the ileum was used, and the measurement was extended from the mesenteric till the antimesenteric border. The mean value of the two measurements represented the width of the harvested ileum. The values were inserted in an IRB-approved customised database (PTV registration number 263.22).

### 2.1 Geometric hypotheses for modelling

A mathematical theoretical analysis was performed to evaluate the impact of ileal width on the pouch capacity. To confirm the thesis of significant variations in volume in function of the width of the bowel, we made use of a geometric model in which the following hypotheses were assumed:

1. All the tissue (surface) of the ileum is utilised in the pouch; the tissue incorporated in the suture lines is of a negligible surface area.
2. The shape of the pouch is a spheroidal.
3. The harvested ileum is a cylinder with a constant diameter.
4. The thickness of the bowel is negligible.

With these assumptions, a mathematical formula was created, which allowed to calculate (1) for a given length of harvested ileum, the variability in pouch capacity in function of the ileal width, and (2) for a given width of the patient's small intestine and a desired volume *V* of the ONB to be created, the necessary length *L* of the intestine to harvest.

**Fig. 1:**
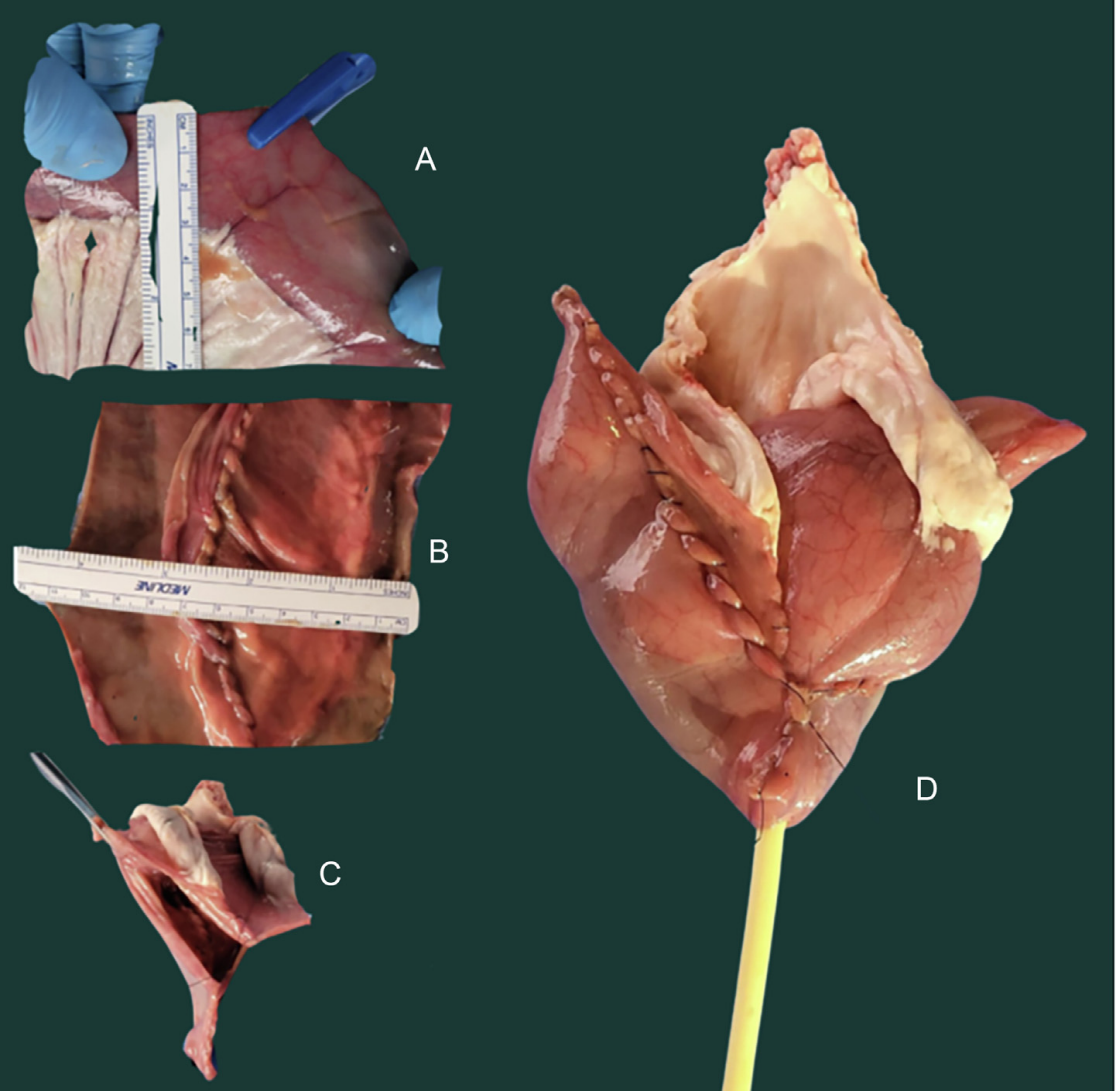
(A) Measurement of the width of the selected ileal segment for the closed ileum. Two measurements are performed, at 10 cm from the ends of the cylinder, and the mean value is calculated. In this case, the mean width was 2.8 cm (3 and 2.5 cm, respectively). (B) The new measurement after the creation of the posterior plate confirms that, although the width of the opened ileum is slightly increased in comparison with the closed cylinder due to the ileal thickness, the extra width is incorporated in the suture line. Thus, the final width of the posterior plate corresponds to four times the width of the closed ileum. (C) Pouch reconstruction according to the principles of the Bordeaux neobladder in order to obtain a spherical reservoir. (D) Catheterisation and final aspect of the pouch.

The accuracy of the developed model to predict the pouch capacity was tested in 12 40-cm segments of different swine ilea obtained from a local slaughterhouse from animals destined for alimentary purposes. Immediately after slaughter, the tissue was rinsed with distilled water to remove blood and body fluids, then transported in a saline solution (0.9% w/v NaCl). The width of the harvested ileum was measured as described previously (Fig. 1). The middle antimesenteric part of the ileal segment was opened, and the width of the opened ileum was measured again. Following detubularisation, the posterior and anterior reconstructions were performed according to the principles of the Bordeaux neobladder (Fig. 1) [9], [10]. After the creation of the posterior plane (with the incorporation of a small area of tissue in the suture line), a final measurement of its width was performed. The maximum time of tissue permanence in saline was 2 h for every pouch.

**Fig. 2:**
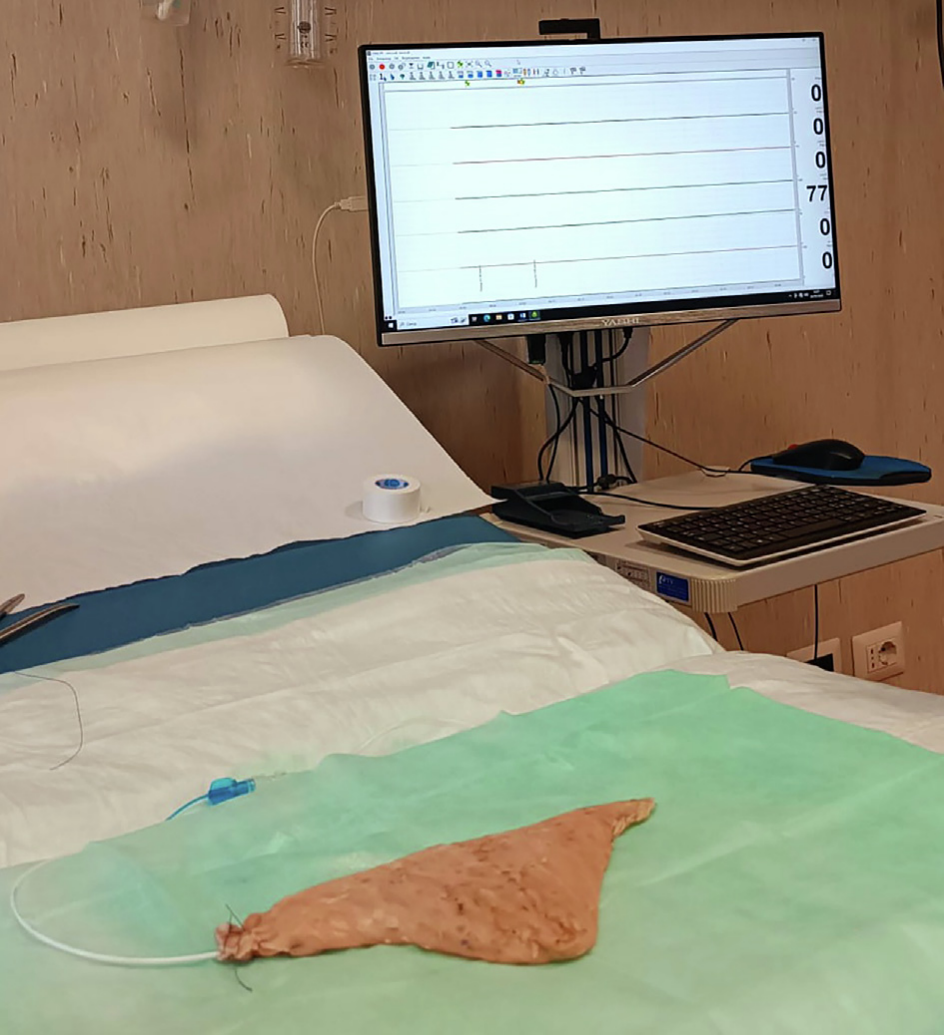

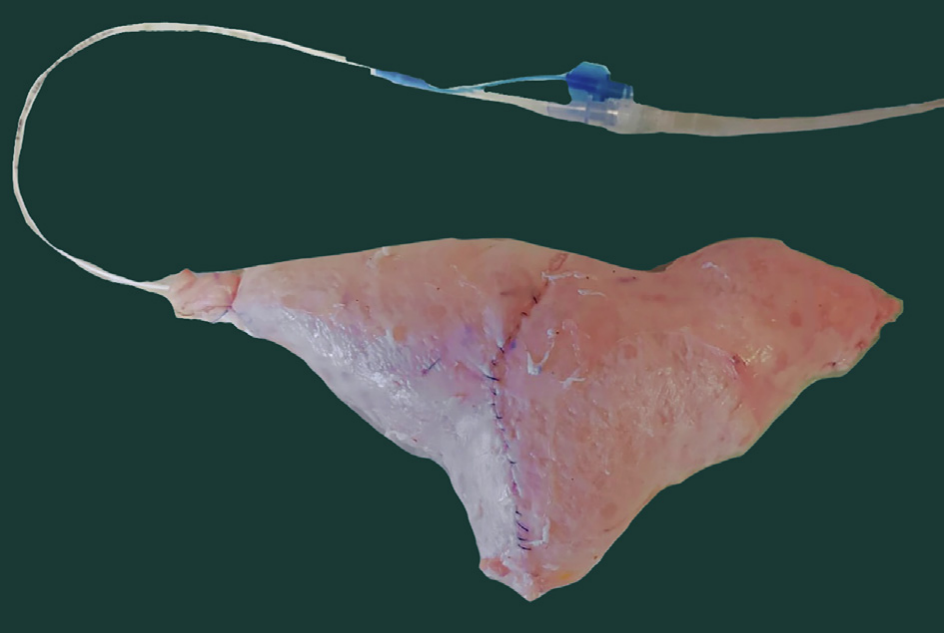
(A and B) Room set-up for the study of the pouch capacity.

The mathematical formula was then tested for discrepancies between the predicted and effective volumes of the pouch. In six cases, a Foley catheter was inserted in the reservoir; a stitch around the catheter at the level of the reservoir neck was ligated to prevent leaks, and the pouch was then filled with saline solution. In six cases, the filling was performed by a dual-lumen 7F urodynamic catheter at a rate of 50 ml/s with normal saline, with simultaneous registration of the pressure inside the reservoir (Fig. 2A and 2B). In both cases, the maximum capacity was defined as a saline leak from any of the suture lines (after a preliminary test to correct any leak due to untight suture), following the principles of the cystographic measurement of bladder capacity.

**Supplementary Fig. 1:**
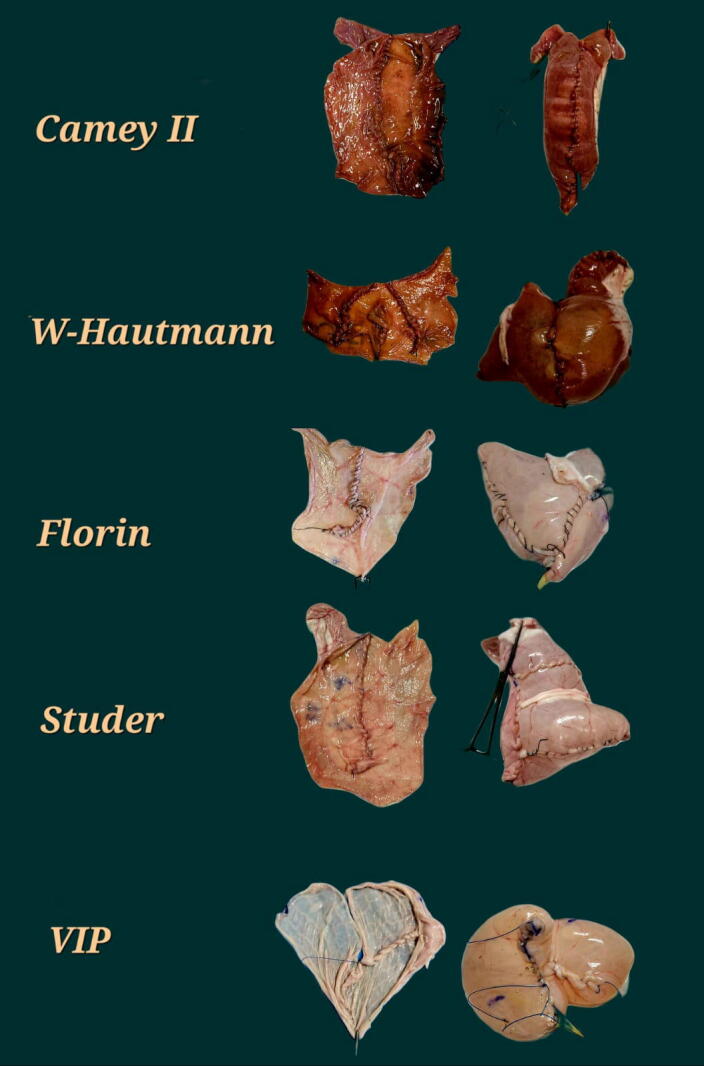
Tests between formula-calculated and effective pouch capacity on pouches created according to the principles of the Studer, Hautmann, VIP, Florin and Camey II.

Similar tests between formula-calculated and effective pouch capacity were performed in ten more pouches that were created according to the Studer, Hautmann, VIP, Florin, and Camey II principles (Supplementary Fig. 1).

As a second step, the width of the isolated ileum was measured in preterminal ileal segments of six more swine. With a target capacity of 350 ml, the formula was used to calculate the necessary ileal length to harvest to obtain this capacity. The Bordeaux technique was used to create the pouch using the calculated length of the ileum. Once configured, the capacity of the reservoir was measured as described previously.

Data were analysed using descriptive statistics. Proportions were used for categorical variables, and means and standard deviations were used for continuous variables. Pearson correlation coefficient was calculated to evaluate the relationship between formula-calculated and effective pouch capacity.

## 3 Results

### 3.1 Variability in the human (pre)terminal ileum width

The mean hemicircumference of the human distal ileum was 2.43 ± 0.39 cm, with a range of 2–3.5 cm.

### 3.2 Modelling—the neobladder AADAPT formula

The supplementary material summarises the variables that were taken into consideration for the geometric model and describes step by step the mathematical development of the model itself. The final formula is as follows:

$$L=\frac{\sqrt[{3}]{\frac{9}{2}\pi V^{2}}}{w}$$

As demonstrated, for the same desired capacity (volume), the length of the ileal tube is inversely proportional to its width.

Examples are provided in the following sections.

#### 3.2.1 Definite length

**Fig. 3:**
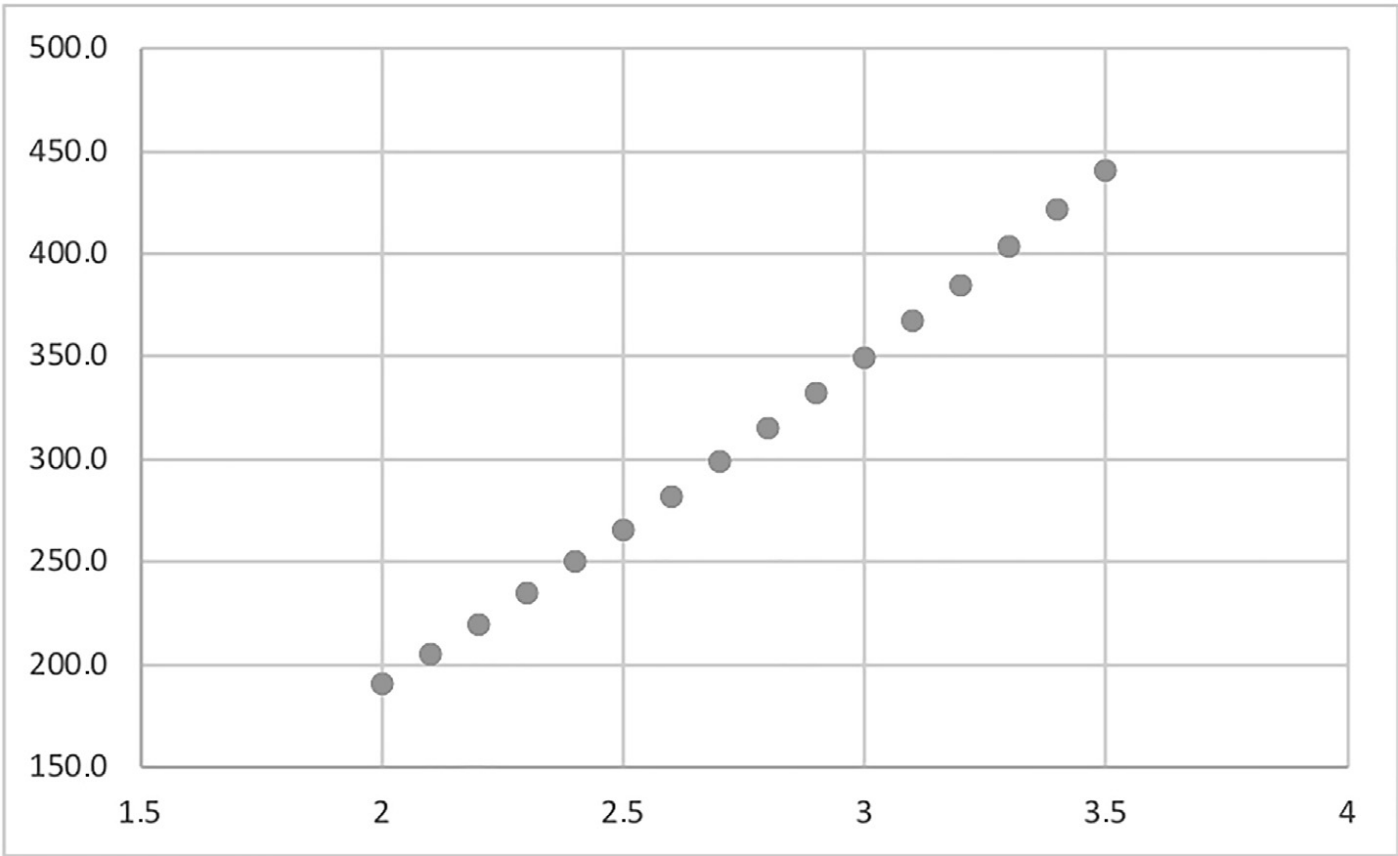
Variability of the pouch volume in function of the ileal width, for a given ileal length (40 cm). As shown, variations of a few millimetres of the ileal width determine great variations in the obtained volume (capacity). For example, for an ileal width of 2 cm, the functional capacity of the ONB is 190 ml, while for 3 cm, the capacity reaches 350 ml. ONB = orthotopic neobladder.

Figure 3 reports the variability of the ONB volume in function of the ileal width, for a given ileal length.

As shown, variations of a few millimetres of the ileal width determine great variations in the obtained volume (capacity). For example, for an ileal width of 2 cm, the functional capacity of a 40-cm pouch is 190 ml, while for 3 cm, the capacity increases to 350 ml.

**Fig. 4:**
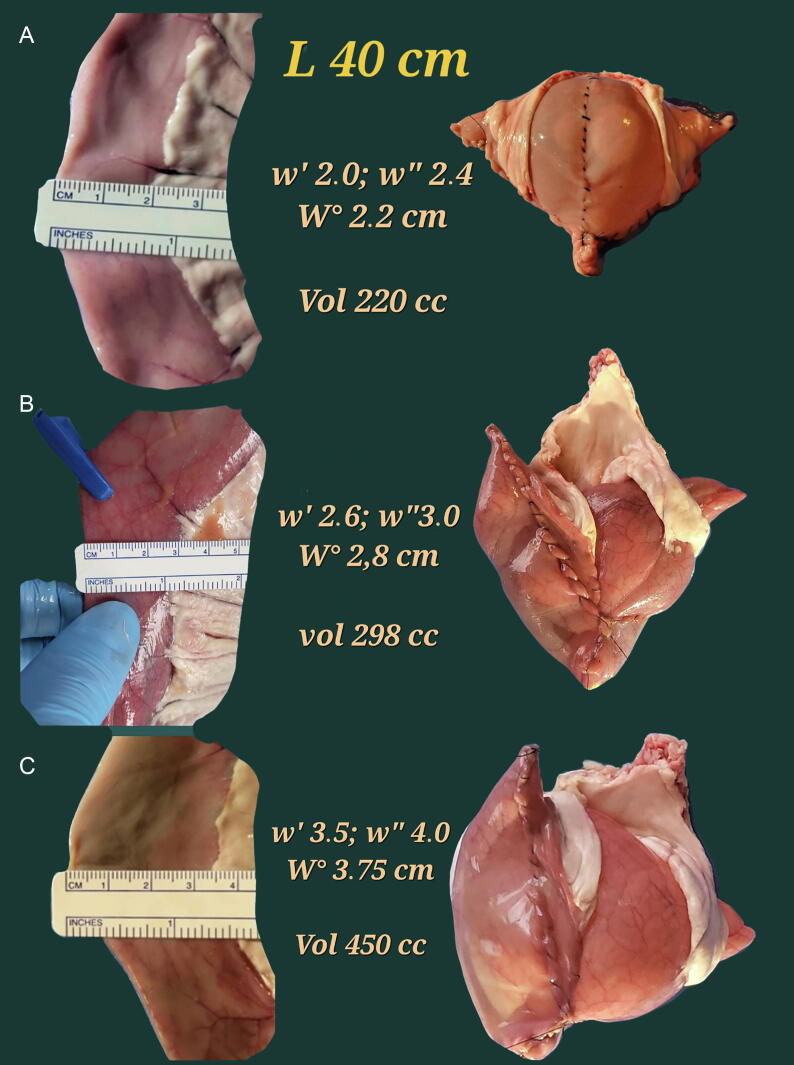
Swine ileal pouches according to the Bordeaux technique, same length of ileum, different width. (A) For a mean ileal width of 2.2 cm, the pouch effective capacity is 220 ml. (B) For a mean ileal width of 2.8 cm, the pouch effective capacity is 298 ml. (C) For a mean ileal width of 3.75 cm, the pouch capacity is 450 ml.

Figure 4 shows the variability in capacity for pouches created by 40 cm of swine ileum in function of the ileal width.

**Fig. 5:**
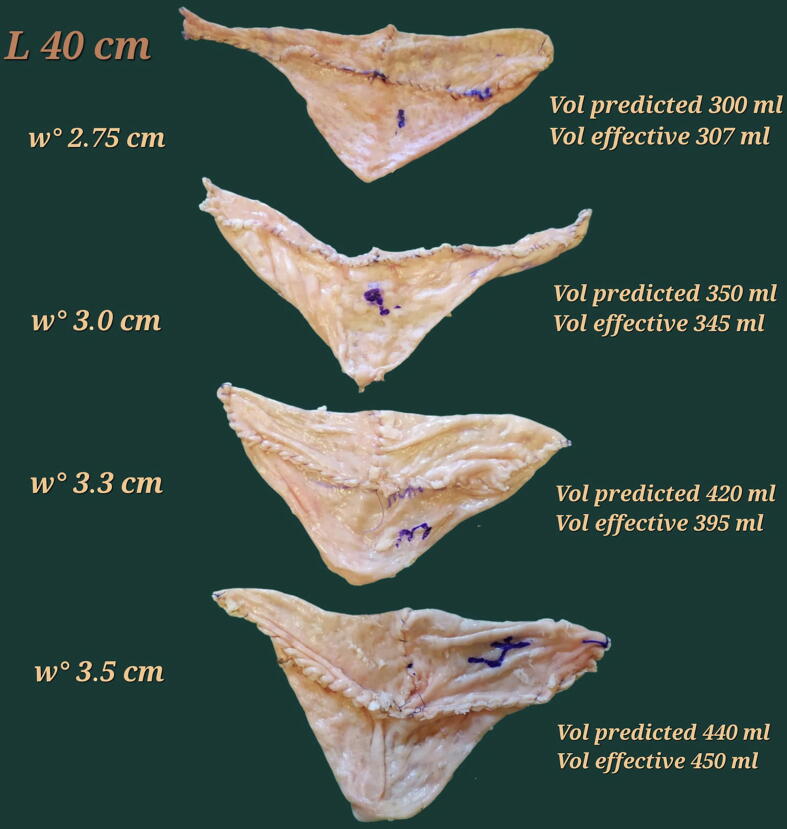
Bordeaux pouches, created with the same ileal length of 40 cm. The difference in the capacity is visibly appreciated, although the same length and shaping of the pouch have been adopted. On the left, the mean ileal width of the selected ileal segment for the closed ileum, and on the right, the formula-predicted and the effective capacity of the pouch are shown. As shown, the formula provides accurate prediction of the capacity.

The test on the swine model did not document significant variations between formula-calculated and effective capacity, as shown in Figure 5.

**Fig. 6:**
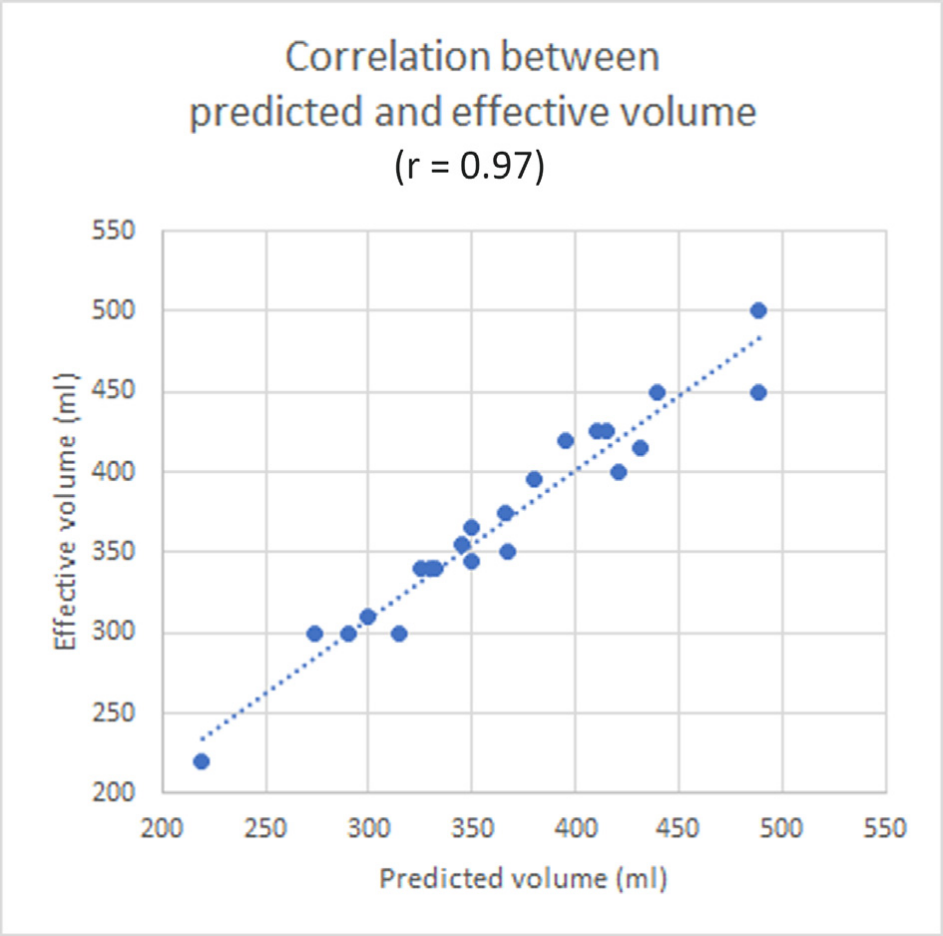
Scatter plot graphically representing the correlation between formula-calculated and effective pouch capacity. Pearson’s correlation coefficient demonstrated a strong positive relation between the formula-calculated and effective pouch capacity (*r* = 0.97).

The differences between formula-calculated and effective pouch capacity are graphically depicted by a scatter plot (Fig. 6). Pearson’s correlation coefficient demonstrated a strong positive relation between formula-calculated and effective pouch capacity (*r* = 0.97).

#### 3.2.2 Definite volume

**Fig. 7:**
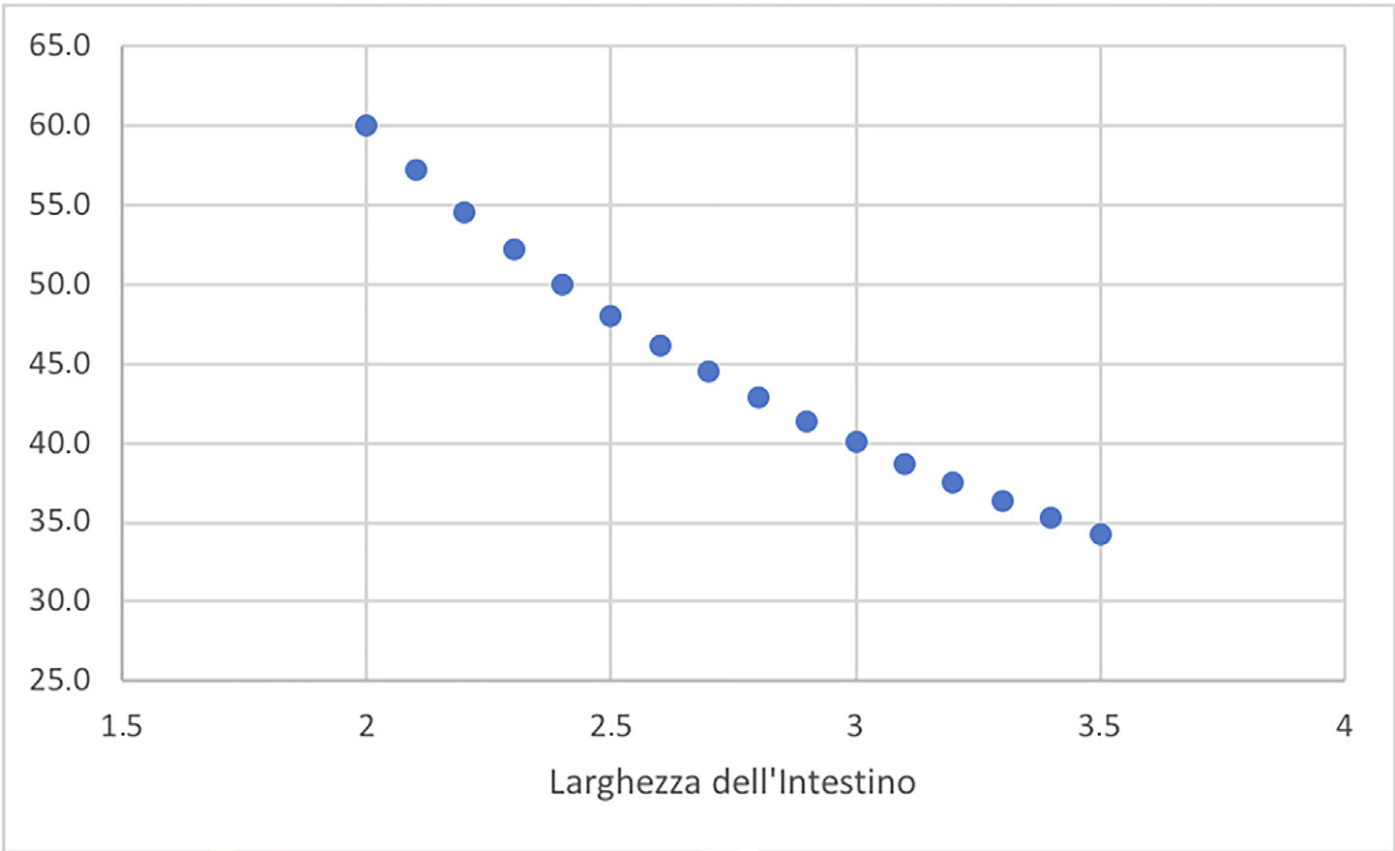
Variability of the ileal length to harvest in function of the ileal width, for a desired ONB capacity. For example, for the same desired capacity (350 cc) of the ONB, if the ileal width is 3.5 cm, then only 35 cm of ileum should be harvested, compared with the 60 cm that is required if the width is 2 cm. ONB = orthotopic neobladder.

Assuming an ONB volume of 350 ml as a target, Figure 7 summarises the length of the ileum to harvest as a function of the ileum width.

According to this geometric model, for the same volume to be obtained, just a few millimetres of difference in the width of the intestine imply a significant difference of several centimetres in length to harvest. In our example, for a desired capacity of 350 ml, 60 cm of ileum is required if the width is 2 cm compared with only 35 cm if the ileal width is 3.5 cm.

**Fig. 8:**
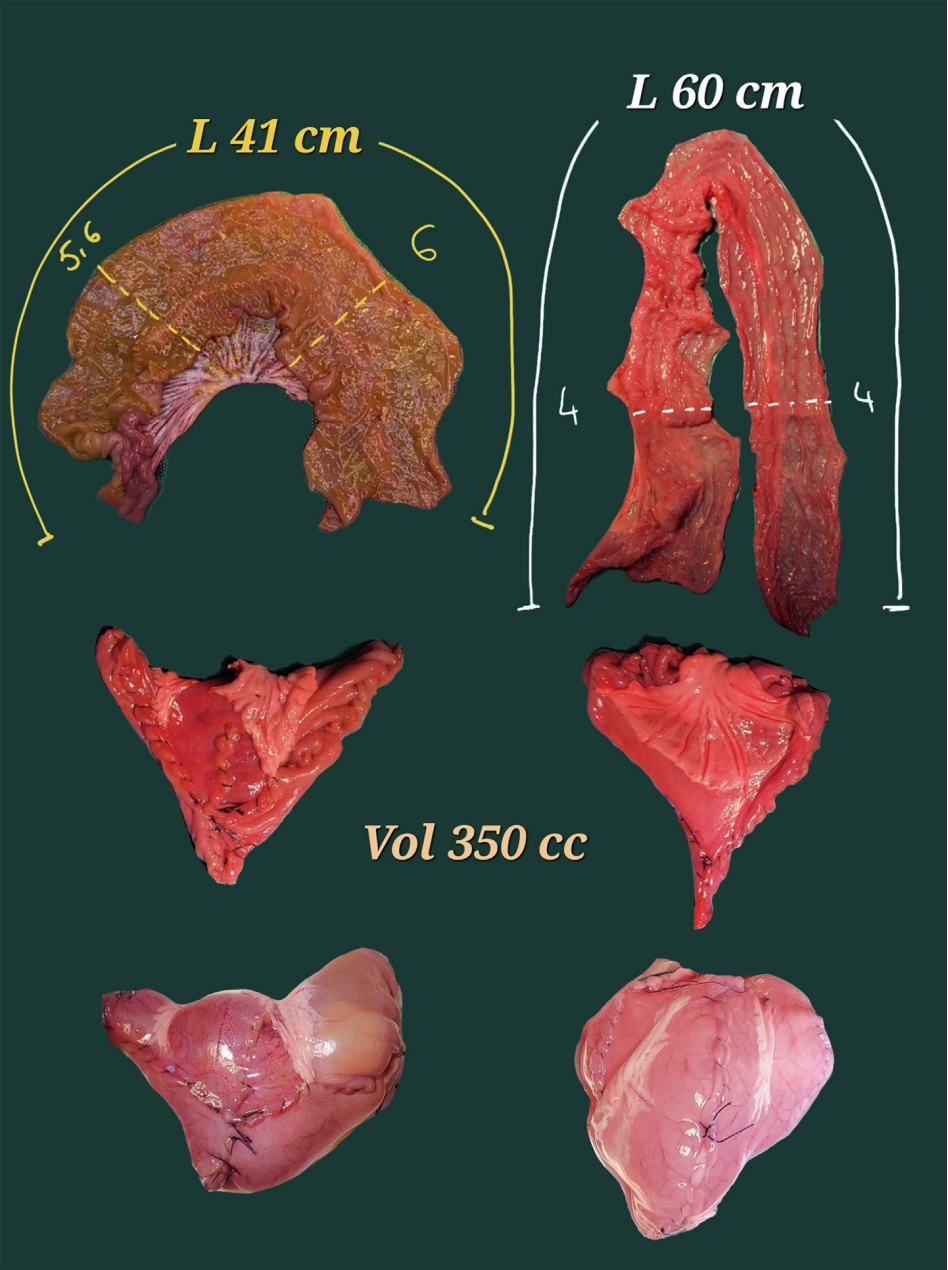
Swine ileal pouches according to the Bordeaux technique, same target capacity, different width. For a mean ileal width of 2.9 cm and for a target capacity of 350 ml, 41 cm of ileum was harvested, as indicated by the formula (left side). For a mean ileal width of 2 cm, 60 cm of ileum was harvested (right side). In both cases, the obtained pouch had the same capacity, that is, 350 ml.

The test on the swine model proved that when the length of harvested ileum is tailored on the width, the formula-calculated pouch capacity is the same, as shown in Figure 8.

In each case, the width of the opened ileum was 1.9 ± 0.48 mm superior to the hemicircumference of the closed cylinder because of the inclusion of the ileal thickness into the measurement. However, after the creation of the posterior plane with the incorporation of ileal tissue in the suture line, the overall width of the posterior plate corresponded to four times the width of the closed tube.

## 4 Discussion

An ileal ONB is an option for UD after radical cystectomy for bladder cancer in men and women [4], [12].

When constructing an ileal ONB, modifications primarily include variations in the length of the harvested ileum, the exact folding technique (based on the type of ONB to be created), and the management of the ureters [13], [14]. In each case, the aim is to create a spherical reservoir, since it represents the configuration that has the most volume for the least surface area. By increasing the volume, it has been suggested that pressure relationships within the reservoir are reduced. This is based on Laplace’s law, which states that for a sphere, the tension of its wall is proportional to the product of the radius and pressure. Thus, theoretically, for a given wall tension, the greater the radius, the smaller the generated pressure. This is desirable in an attempt to prevent deterioration of the upper tracts or incontinence.

In all the techniques described, the efforts to increase the radius of the reservoir are based on the increases of the length of the harvested ileum or the number of ileal folding. In fact, ileal reservoirs may use from 40 to 75 cm of a terminal ileum, which is detubularised and folded in a variety of ways to attempt to create a spherical shape. Then the ureters are implanted with or without an antireflux mechanism.

However, even among cases of the same shape of ONB, great variability in MCC is observed. In fact, for a given type of ONB (and consequently for a definite length and shape of the ileum), significant variations in the obtained functional capacity of the ONB are observed [5], [6], [7], [8], [9], [10]. A common technical issue in all these techniques is that no consideration is given to the interindividual variability of the width of the ileum.

To our knowledge, this is the first study where the impact of the ileum width on the capacity of the ONB is evaluated. We identified interindividual variability of the ileal width ranging between 2 and 3.5 cm. This range may be the basis of the observed differences of the ONB capacity: variations of a few millimetres of the ileal width determine great variations in the obtained ONB volume (capacity). For example, for an ileal width of 2 cm, the functional capacity of the ONB is 190 ml, while for 3 cm, the capacity reaches 350 ml.

Another indirect demonstration of the impact of the ileal width on the ONB capacity comes from the study of Montie et al. [15]. The authors have compared the W-stapled ileal reservoir with hand-sewn reservoirs through urodynamic evaluation, comparing capacity, maximum pressure at capacity, residual urine, and noninhibited peristaltic contractions during the filling phase. The W-stapled reservoir did not appear to provide as good results as hand-sewn reservoirs made from ileal segments. In fact, six of the 19 evaluable patients with stapled reservoir had poor reservoir characteristics, characterised by smaller mean ONB capacity and higher intraluminal pressure, with three of these patients needing an augmentation cystoplasty. The authors attributed these differences to ischaemia of the ileum between the adjacent stapled lines, which could induce fibrosis and retraction/lack of distensibility of the reservoir. Furthermore, septations persisted in the reservoir at sites where adjacent limbs were not anastomosed entirely (ie, lack of full detubularisation), which could have interfered with the spherical shape. However, another possible factor may be represented by the reduction in the ileal width due to the tissue (surface) that is included between the staple lines. In fact, the tissue incorporated in the staple lines, contrary to the running suture, is not of a negligible surface area. The use of the staples may consequently result in a shorter pouch radius, leading to decreased pouch compliance and lower functional capacity.

In this study, we also assume that for a given type (shape) of ONB, no standard length of ileum should be harvested; instead, the length should be tailored to the width of the ileum for a given patient. Our mathematical formula calculates, for desired ONB capacity and a given ileal width (that is not modifiable), the length of the ileum to harvest. The model, through geometric simplifications, tries to express the quantitative link that exists between the width of the patient's intestine, the length of the harvested segment, and the volume of the obtained neobladder. For the same shape of ONB with the same target capacity (350 cm), if the ileal width is 3.5 cm, then only 35 cm of ileum should be harvested, compared with the 60 cm that is required if the width is 2 cm, leading to a spare of 25 cm of ileum.

The geometric model was not developed for a particular type of ONB but for any spherical neobladder. The assumptions (1–4) used for the creation of the model aim to create a simple formula unifying the characteristics of the neobladder. In particular, the model does not take into account the technique used for the neobladder reconstruction. All the techniques aim at reducing the overlapping areas of tissue along the sutures, coherently with hypothesis (1), and over time the neobladders tend to assume a spheroidal shape, coherently with hypothesis (2).

Although reality may be far from the assumed geometric hypotheses, the animal tests seem to confirm the values predicted by the model. Thus, the simplifications imposed by the model do not alter the description of the result. It is therefore possible to neglect a series of excessively complex physical and clinical parameters to implement in the formula without altering its effectiveness.

With these premises, first the formula was created and then tested on the spheroidal-shaped Bordeaux neobladder. We preferred to evaluate the accuracy of the formula using only one configuration of reservoir (but representative of all the spheroidal ones) in order to be able to correct the formula in case of significant differences between the effective and formula-calculated volumes without the added bias of multiple configurations. Once we demonstrated that there were no significant differences in these parameters for one type of neobladder, the formula was tested for other spherical reservoirs confirming its accuracy.

It is well known that the volume capacity of an ONB usually increases over time [16], [17]. Thus, the starting ONB capacity is of paramount importance. Our model could be fundamental for the geometric planning of the ONB and may avoid the creation of large hypocontractile reservoirs or small ones with high internal pressure. Further urodynamic studies may aid in its implementation with coefficients that take into consideration the patterns of ONB capacity increase over time, increasing its accuracy.

The current study lacks in prospective testing on humans. Furthermore, the model was created and it is better applied to reservoirs with a spheroidal shape. It does not include parameters that consider the increase of volume capacity of the reservoir over time.

## 5 Conclusions

The interindividual variability in ileal width significantly affects the capacity of an ONB; thus, this parameter cannot be neglected during the ONB reconstruction. Our model allows for predicting the ONB capacity for a given ileal length and calculating in advance the length of the small intestine to harvest according to its intraoperatively measured width, in order to obtain an ONB with the desired volume.

  ***Author contributions:*** Anastasios D. Asimakopoulos had full access to all the data in the study and takes responsibility for the integrity of the data and the accuracy of the data analysis.

  *Study concept and design*: Asimakopoulos, Annino.

*Acquisition of data*: Asimakopoulos, Annino.

*Analysis and interpretation of data*: Asimakopoulos, Annino, Ghattas, Piechaud, Gaston.

*Drafting of the manuscript*: Asimakopoulos, Annino, Ghattas.

*Critical revision of the manuscript for important intellectual content*: Asimakopoulos, Annino, Ghattas, Piechaud, Gaston.

*Statistical analysis*: None.

*Obtaining funding*: None.

*Administrative, technical, or material support*: None.

*Supervision*: Piechaud, Gaston.

*Other*: None.

  ***Financial disclosures:*** Anastasios D. Asimakopoulos certifies that all conflicts of interest, including specific financial interests and relationships and affiliations relevant to the subject matter or materials discussed in the manuscript (eg, employment/affiliation, grants or funding, consultancies, honoraria, stock ownership or options, expert testimony, royalties, or patents filed, received, or pending), are the following: None.

  ***Funding/Support and role of the sponsor:*** None.

  ***Acknowledgements:*** We thank Dr. Lorenzo Dutto, Urologist in the Department of Urology of the Queen Elisabeth’s University Hospital (Glasgow, UK), for his kind help in the linguistic revision of the manuscript.
