# Supplementary material for: Not Only Length Matters! Impact of the Ileal Width on the Capacity of the Orthotopic Neobladder: The AADAPT Formula Tested on the Animal Model
Source: Eur Urol Open Sci. 2023 Nov 8;58:55–63. doi: 10.1016/j.euros.2023.10.003 (PMC10751539; doi:10.1016/j.euros.2023.10.003)
Supplement: Supplementary Data 1 [file mmc1.docx]

| V = volume of the pouch to be constructed |
| --- |
| r = radius of the pouch to be constructed |
| Sn = surface area of the pouch to be constructed |
| L = length of the small intestine segment to be harvested |
| w = width of the harvested intestine, i.e. the hemicircumference (mean of the two measurements) |
| St = surface of the harvested ileal segment |

Starting from hypothesis i), we assume St=Sn. With hypotheses ii) and iii), this becomes 2Lw=4πr^2^, that is $L=\frac{2\pi r^{2}}{w}$

In order to know the radius of the neobladder as a function of its volume, we have, according to hypotheses ii) and iv), $V=\frac{4}{3}\pi r^{3}$

from which the sought radius is obtained $r=\sqrt[3]{\frac{3}{4\pi}V}$.

By inserting this result in the formula of the previously obtained length, we have $L=\frac{2\pi r^{2}}{w}=\frac{2\pi\left( \sqrt[3]{\frac{3}{4\pi}V} \right)^{2}}{w}$

and finally:

$$L=\frac{\sqrt[3]{\frac{9}{2}\pi V^{2}}}{w}$$

- Concerning issue i) (All the tissue (surface) of the ileum is utilized in the pouch; the tissue incorporated in the suture lines is of negligible surface area) and iv) (The thickness of the bowel is negligible) we precise that in our experiment, we first measured the width of the closed ileum, then we measured it again once detubularised. The width of the opened ileum was 1.9±0.48mm superior to the hemicircumference of the closed cylinder because of the inclusion of the ileal thickness to the measurement. However, after the creation of the posterior plane with the incorporation of ileal tissue in the suture line the overall width of the posterior plate corresponded to times four the width of the closed tube; this indicates that the extra width that is due to the ileal thickness is counterbalanced by its incorporation in the suture line; thus, it does not affect the relation between the obtained volume capacity and the calculated one.
- Considering issue iii) (The harvested ileum is a cylinder with constant diameter) we underline that for each ileal segment two measurements at 10cm from each end were performed and the mean value was calculated and used as width (w).
